# Phosphatidylcholine-Based Nanoemulsions for Paclitaxel and a P-Glycoprotein Inhibitor Delivery and Breast Cancer Intraductal Treatment

**DOI:** 10.3390/ph15091110

**Published:** 2022-09-06

**Authors:** Giovanna Cassone Salata, Luciana B. Lopes

**Affiliations:** Department of Pharmacology, Institute of Biomedical Sciences, University of São Paulo, Sao Paulo 05508-000, Brazil

**Keywords:** nanoemulsion, intraductal, breast cancer, paclitaxel, P-gp inhibition

## Abstract

In this study, incorporation of the cytotoxic agent paclitaxel and the P-glycoprotein inhibitor elacridar in hyaluronic acid (HA)-modified nanoemulsions was studied for intraductal delivery and breast cancer localized treatment. To improve cytotoxicity, we investigated the incorporation of perillyl alcohol or tributyrin as components of the nanoemulsion oil phase. The nanoemulsions presented size <180 nm and negative zeta potential. Both tributyrin and perillyl alcohol increased nanoemulsion cytotoxicity in MCF-7 cells, but not in MDA-MB-231. However, perillyl alcohol reduced nanoemulsion stability in the presence of the drugs. Concomitant incorporation of paclitaxel and elacridar in HA- and tributyrin-containing nanoemulsions (PE-NETri) increased cytotoxicity and reduced IC_50_ by 1.6 to 3-fold in MCF-7 and MDA-MB-231 cells compared to the nanoemulsion containing only paclitaxel (P-NE). This nanoemulsion also produced a 3.3-fold reduction in the viability of MDA-MB-231 spheroids. Elacridar incorporated in the nanoemulsion was capable of inhibiting P-glycoprotein in membranes. In vivo intraductal administration of the NE containing HA resulted in a three-fold higher retention of a fluorescent marker compared to a solution or nanoemulsion without HA, demonstrating the importance of HA. The nanoemulsion produced no histological changes in the mammary tissue. These results support the potential applicability of the nanoemulsion for local breast cancer management.

## 1. Introduction

Ductal carcinoma in situ (DCIS) consists of neoplastic lesions in the ductal-lobular structures of the breast without invading the basal myoepithelial membrane, and comprises 25% of the diagnosed breast cancer cases [1]. Because its progression to invasive forms can occur and no prediction strategies for this transformation are currently available, DCIS management usually follows an aggressive pattern, involving surgery with or without breast conservation and long-term endocrinotherapy and radiation [2]. Since DCIS develops within the ducts, drug delivery directly into the ducts is useful to maximize local drug concentration and minimize systemic adverse effects. Intraductal drug delivery has been proposed in preclinical and clinical studies as a localized strategy to treat breast cancer and to limit and/or reverse the carcinogenesis process, also serving for chemoprevention in high-risk patients [3,4,5,6].

Due to the ductal permeability, previous evidence suggested that small drugs are likely to diffuse into the systemic circulation after intraductal administration as solutions, although to a lesser extent compared to systemic administration [7], which can be problematic considering the high toxicity of antitumor drugs. In addition to ductal permeability, its cannulation for delivery requires trained personnel. Thus, the use of strategies to improve ductal retention of drugs is necessary to combine efficacy, safety, and reduce the frequency of administration [8]. Nanocarriers, such as nanoemulsions, lipid nanoparticles, polymeric aggregates, and nanosuspensions have been proposed as delivery systems to fulfill this need [7,8,9,10]. Singh et al. demonstrated that increases in the molecular weight of PEG-polymer aggregates increased retention in the ducts [7]. Ductal retention of micro and nanoparticles have also been demonstrated, with 1 μm particles providing longer retention than 100 and 500 nm nanoparticles [11]. Administration of an in situ gel also provided longer retention times of a fluorescent dye compared to its solution, suggesting the importance of viscosity and bioadhesion for local retention [11]. Our group has previously demonstrated that nanoemulsions containing bioadhesive polymers prolonged the retention of fluorescent markers in the mammary tissue compared to solutions [8,12].

In the present study, hyaluronic acid (HA)-modified nanoemulsions containing cytotoxic additives as the oil phase were designed for co-incorporation and intraductal delivery of paclitaxel and elacridar. Nanoemulsions are colloidal dispersions of two immiscible liquids (most often oil and water) stabilized by surfactants. Nanoemulsions were selected as delivery systems due to several properties and advantages over other nanocarriers: (i) The oil phase aids solubilization of lipophilic compounds such as paclitaxel and elacridar while still enabling their dispersion in an aqueous external phase; (ii) the presence of a surfactant interface offers an additional area for incorporation of drugs with poor aqueous solubility; (iii) nanoemulsions can incorporate large amounts of water (>60%) and can be obtained with lower amounts of surfactants compared to other emulsified nanosystems, such as microemulsions, which generally result in a lower irritation potential; (iv) it is possible to modify the surface of the droplets with ligands, improving cell internalization and selectivity, among other properties; and (v) several types of oil phases can be utilized for their production, and their selection can be based on their ability to dissolve the drug and/or to confer additional properties to the formulation (such as an improved cytotoxicity) [13,14,15,16]. Nanoemulsion modification with HA is justified by its bioadhesive properties (to prolong mammary tissue retention) and its affinity for the differentiation cluster protein 44 (CD44), a receptor overexpressed in several types of cancer, which has been suggested to aid cancer cell targeting [17]. We hypothesized that these nanoemulsions could prolong mammary tissue retention, potentiate paclitaxel cytotoxicity, and inhibit P-glycoprotein efflux transporter (P-gp), helping to revert multidrug resistance.

Paclitaxel (P), a well-known cytotoxic drug, has been used in the clinic for treatment of early and metastatic breast cancer, proving to be more effective than doxorubicin and cyclophosphamide [18,19,20]. Its mechanism of action is mainly attributed to its ability to bind to β-tubulin, stabilizing the polymerized microtubule, stopping cell cycle in G_2_/M and apoptosis [21,22]. Its specificity against tumor cells is low, which results in many adverse effects when systemically administered and justifies its choice for direct delivery in the ducts [23]. Furthermore, the efficacy of this drug is limited by tumor resistance, often attributed to overexpression of efflux proteins, with emphasis on P-gp of the ABC family (ATP-binding cassette) [24,25]. In order to circumvent resistance development, elacridar (E) was added to the nanoemulsion. Elacridar is a potent inhibitor of P-gp and breast cancer resistance protein (BCRP), whose advantages include its ability to inhibit P-gp at concentrations 100 times lower than weak inhibitors such as verapamil, without being a substrate for it [26]. We have previously observed that elacridar co-incorporation in nanoemulsions with P-gp substrates promotes drug retention in the epidermis, demonstrating that elacridar incorporation did not preclude its ability to inhibit the efflux transporter expressed in the skin [27].

To further improve the nanoemulsion cytotoxicity, perillyl alcohol and tributyrin were studied as additives of the nanoemulsion oil phase. There is evidence that perillyl alcohol controls cell proliferation and causes cell death without affecting normal cells by the isoprenylation of ras gene products and inhibition of the modulating function of the nuclear factor kappa B (NF-κB) expression [28,29,30]. Tributyrin is a prodrug of butyric acid and reported as a possible anticarcinogenic agent through caspase-3-dependent or -independent pathways involving the down-regulation of *Bcl-2* and up-regulation of *Bax* [31]. To support a possible role of tributyrin on cytotoxicity enhancement, incorporation of C6 ceramide in tributyrin-loaded nanoemulsions increased its cytotoxicity against MCF-7 cells [8].

In the first part of this study, nanoemulsion composition was optimized, and the influence of composition on physicochemical properties, nanoemulsion stability and cytotoxicity was investigated. Subsequently, nanoemulsion effects on P-gp inhibition and paclitaxel cytotoxicity were studied in 2D and 3D breast cancer models using MCF-7 and MDA-MB-231 cells, which were selected for their distinct expression of estrogen receptor (ER), progesterone receptor (PR), human epidermal growth factor receptor 2 (HER2), and CD44. MCF-7 cells are ER^+^, PR^+^, HER2^+^, and CD44^+^, being frequently employed as in vitro models for breast cancer as they mimic between 79% and 84% of the cases [32]. MDA-MB-231 cells are ER^-^, PR^-^, HER2^-^, and CD44^+++^, and have been largely employed as models for triple negative breast cancer [33]. Having an improved cytotoxicity against MDA-MB-231 cells is relevant due to the lack of effective treatment options for triple negative breast cancer [34,35]. Finally, the in vivo ability of a selected formulation to prolong local retention was assessed.

## 2. Results

### 2.1. Nanoemulsion Development, Composition Selection, and Cytotoxicity

In the first part of this study, we compared the effects of various concentrations of perillyl alcohol (PA), tributyrin (Tri), and hyaluronic acid (HA) on nanoemulsion characteristics. To assess the effects of Tri and PA, three types of oil phase were tested: tricaprylin and tricaprylin containing either perillyl alcohol or tributyrin (ranging from 0.5 to 5% as final concentration in the nanoemulsion). HA was dissolved in PBS (prior to the aqueous phase addition to the surfactant:oil phase mixture) to obtain final concentrations of 0.125, 0.25, and 0.5%. The goal was to optimize the nanoemulsion composition by maximizing the content of PA, tributyrin, and HA while maintaining nanoemulsion stability. After defining the composition, selected nanoemulsions were subjected to a comparison of their cytotoxicity in cell monolayers.

#### 2.1.1. Influence of the Oil Phase on NE Characteristics: Effects of PA and Tributyrin

We first assessed the impact of the oil phase on the nanoemulsion characteristics. For this evaluation, nanoemulsions did not contain HA. At 1 and 2.5%, PA enabled formation of nanoemulsions with droplet diameter below 200 nm and PDI <0.21, denoting a narrow size distribution (Table 1). However, the NE containing 2.5% of PA became more viscous after 5 days, which could make its intraductal administration challenging. Increasing PA concentration to 5% hindered the formation of fluid systems, resulting in a viscous emulsion instead. Similarly, viscous emulsions were obtained when PA was replaced by tributyrin at 2.5 or 5%; only at 1% tributyrin enabled the formation of fluid systems with droplets of ~100 nm (Table 1). All nanoemulsions displayed negative zeta potential, with the formulation containing tributyrin (NETri without HA) presenting the lowest value. These results suggest that PA or tributyrin content over 1% precluded the obtainment of nanoemulsions with the desired characteristics for intraductal use.

Under a polarized light microscope, the nanoemulsions containing 1% of tributyrin or PA did not display any specific textures that could be associated with lamellar phase formation, large droplets, or aggregates. Because the macroscopic and microscopic appearance of these nanoemulsions did not change for 7 days, they were subjected to HA incorporation.

#### 2.1.2. Influence of HA Incorporation on NE Characteristics

**Figure 1:**
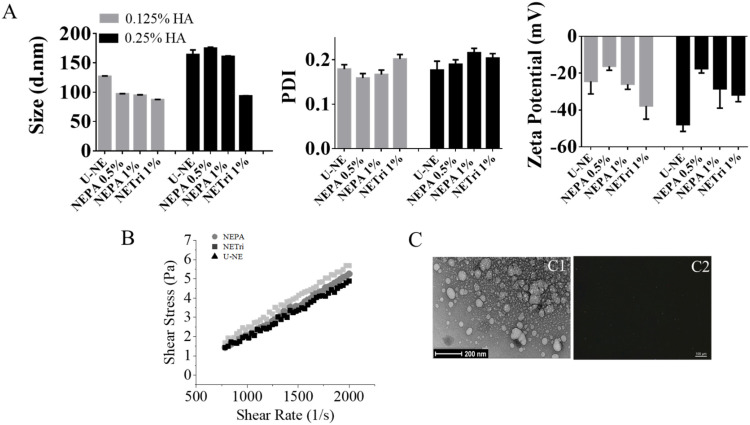
Nanoemulsion development and characterization. (**A**) Influence of NE composition (unloaded, containing perillyl alcohol or tributyrin) and HA concentration on the size distribution, polydispersity index (PDI), and zeta potential. (**B**) Rheological behavior of U−NE (NE containing only tricaprylin as oil phase and 0.25% HA), NEPA (NE containing tricaprylin with 0.5% PA and 0.25% of HA), and NETri (tricaprylin with 1% tributyrin and 0.25% HA). (**C**) Microscopy images of NETri. (**C1**) Representative image of transmission electron microscopy of NETri. (**C2**) Representative image of polarized light optical microscopy of the formulation. At least three batches of each formulation were produced.

HA was included at 0.125–0.5% to improve bioadhesive properties and mammary tissue retention. At 0.5%, HA did not enable the formation of homogeneous nanoemulsions. At 0.125 or 0.25% of HA, all formulations containing only tricaprylin as oil phase (U-NE), tricaprylin with PA (NEPA1%) or tributyrin (NETri1%) at 1% (*w/w*) displayed diameter lower than 180 nm, PDI < 0.22 and negative zeta potential (Figure 1A). However, after 7 days, creaming was observed in the formulations containing 1% PA, but not tributyrin. Upon shaking, the formulations returned to their original aspect, but an increase in particle size and PDI (above 0.3) was observed, reflecting the lack of stability of the 1% PA-containing formulations in the presence of HA. Reducing PA concentration to 0.5% (NEPA0.5%) resulted in formulations stable for 1 week. Comparing all the nanoemulsions, the increase in HA content to 0.25% did not reduce zeta potential in a significant (*p* > 0.05) manner. Based on these results, HA at 0.25% was selected to increase bioadhesion and three types of nanoemulsions were subjected to further characterization, and cytotoxicity and stability evaluation: NE containing only tricaprylin as oil phase and 0.25% HA (which will be referred to as U-NE), tricaprylin with 0.5% PA and 0.25% of HA (which will be referred to as NEPA), or tricaprylin with 1% tributyrin and 0.25% HA (NETri).

#### 2.1.3. Characterization of Selected Formulations

The selected nanoemulsions were further characterized for rheological behavior (Figure 1B). All formulations presented low viscosity (below 0.005 Pa.s), which is justified by the fact that water represents 80% of their composition. The Newtonian behavior is suggested by the linear relationship between shear rate and shear stress, implying that tributyrin and PA, at the concentrations used, did not affect the rheological behavior of the nanoemulsion, although PA seemed to promote a slight increase in viscosity. A representative image of the nanoemulsion containing tricaprylin is represented in Figure 1(C1). As can be observed, spherical droplets of less than 200 nm were obtained, which corroborates the diameter obtained by light scattering. Similar images were obtained for the other nanoemulsions. No larger droplets or other textures were observed under the polarized light microscope (Figure 1(C2)), suggesting that the use of PC in the surfactant blend did not lead to the formation of liquid crystalline bulk phases [36]. Furthermore, the pH of the formulations (around 6.7, measured by pH strips) suggests compatibility with the planned route [37], since the intraductal route is parenteral and requires a pH as closest to physiological pH as possible to reduce local discomfort [38].

#### 2.1.4. Influence of Nanoemulsion Composition on Its Cytotoxicity

Having optimized the concentration of Tri, PA, and HA in the nanoemulsions, the selected formulations were subsequently subjected to a comparison of their cytotoxicity in cell monolayers. The justification for comparing nanoemulsions with various compositions is based on the facts that (i) HA presents bioadhesive properties and is a ligand of CD44, which might affect interactions with cells and cytotoxicity, and (ii) tributyrin and perillyl alcohol have been described to improve cytotoxic properties of drugs and formulations [8,16,29].

**Figure 2:**
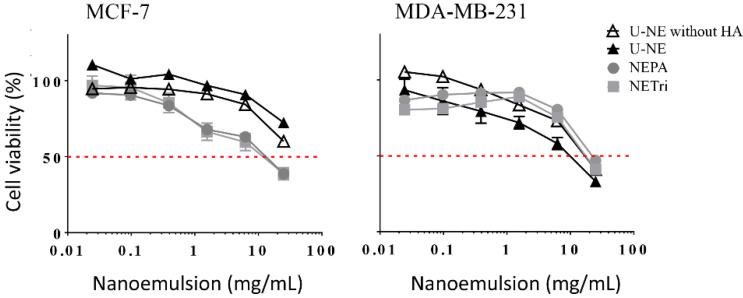
Influence of the concentration of U-NE, U-NE without HA, NEPA, and NETri on the viability of MCF-7 and MDA-MB-231 cells in monolayer (2D culture) after 48 h of treatment. Data are represented as the mean ± SD, *n* = 12–16 in 3–4 separate experiments.

The effect of HA addition was assessed by comparing the NE containing tricaprylin in the oil phase with and without HA at 0.25% (U-NE). The nanoemulsion without HA, tributyrin or PA reduced the viability of MCF-7 cells to ~70% at the highest concentration employed, whereas a similar effect was observed for MDA-MB-231 cells at a lower concentration (Figure 2). The cytotoxicity of unloaded nanoemulsions might be attributed to its components. Surfactants (including polysorbates) and components of the oil phase of emulsified nanocarriers have been demonstrated to affect membrane permeability and increase the release of inflammatory cytokines, and thus, impact cell viability [39,40,41,42]. This is consistent with the previously reported ability of nano and microemulsions to affect the viability of tumor and non-tumor cells depending on the type/concentration of the oil phase and surfactants [43,44,45,46], and might contribute to the overall drug-loaded nanoemulsion cytotoxicity. Presence of HA did not affect the viability of cells in a pronounced manner (Figure 2 and Table 2).

Subsequently, the cytotoxicity of NETri and NEPA (both containing 0.25% HA) was compared to assess the influence of tributyrin and PA addition. These nanoemulsions had comparable cytotoxicity in both cell lines (Figure 2, Table 2), and displayed similar IC_50_ values despite the fact that tributyrin concentration was twice higher. Compared to U-NE, NETri and NEPA had lower IC_50_ values only in MCF-7 cells, which support previous observations that addition of tributyrin to nanoemulsions increase their cytotoxicity in MCF-7 cells [8], and demonstrate that this effect can be achieved at a ~eight-fold lower concentration compared to that previously employed. Because of this cytotoxicity improvement, NETri and NEPA were selected for incorporation of elacridar and paclitaxel.

### 2.2. Influence of Paclitaxel and Elacridar Incorporation on Nanoemulsion Characteristics

At the highest concentration studied (0.1%), elacridar (E) did not enable formation of nanoemulsions with the desired size (Table 1). At this concentration, elacridar was not completely dissolved in the surfactant:oil phase mixtures and few remaining drug crystals were observed under the polarized light microscope, which might justify the increase of ~2.2–6-fold on droplet diameter. Reducing its concentration to 0.07% enabled its solubilization and obtainment of droplets lower than 200 nm (Table 1).

At 1%, paclitaxel (P) solubilization in the surfactant:oil phase mixture (that also contained E at 0.07%) was also very difficult; regardless of the presence of tributyrin or PA, the resulting nanoemulsions displayed PDI >0.4 and sedimentation was observed after 2 days. This high PDI might be related to the presence of two populations (observed in the DLS size distribution) and non-dissolved paclitaxel (Supplementary Figure S1). Reducing paclitaxel concentration to 0.5% resulted in nanoemulsions with narrower size distribution. Inclusion of paclitaxel in NETri (PE-NETri) reduced droplet size by approximately two-fold compared to the nanoemulsions containing only elacridar (E-NE). Although drugs might be able to induce changes on the characteristics of PC-based systems [47], we attribute this reduction to a longer bath sonication (~10 min) for solubilization of paclitaxel, which might have improved the overall formulation homogenization.

We did not measure drug entrapment but worked, instead, with the concept of “drug incorporation in the nanoformulation”. We considered that drug incorporation encompasses the sum of the fractions of drug dissolved/entrapped into the oil droplets, in the surfactant interface, and dispersed/dissolved in the aqueous external compartment due to the presence of propylene glycol (although incorporated in the surfactant blend, it might partition into the aqueous phase), surfactant monomers, and formation of other structures (as micelles due to the excess of surfactant), preventing drug precipitation [48,49,50]. This is supported by previous studies that reported an increase in the apparent solubility of APIs promoted by “liposomalization” and/or “micellization” [51]. Since the selected drug concentrations did not result in precipitation, we considered 100% of incorporation.

**Figure 3:**
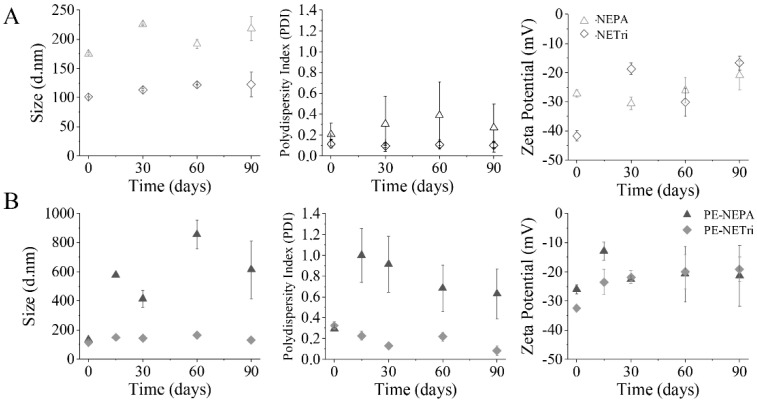
Nanoemulsion short-term stability. (**A**) Influence of NE composition (NEPA and NETri) on size distribution, polydispersity index (PDI) and zeta potential for 90 days. (**B**) Influence of NE loading with paclitaxel and elacridar (PE−NEPA and PE−NETri) on size distribution, polydispersity index (PDI) and zeta potential for 90 days. At least three batches of each formulation were analyzed.

The drug-loaded nanoemulsions were subjected to a short-term stability study (90 days) and compared to the unloaded formulations. All unloaded formulations displayed size below 200 nm, with PDI ranging from 0.11 to 0.20 and zeta potential of −12 to −42 mV at the time of obtainment (Figure 3A). After 90 days, all formulations showed an increase in size: approximately 21.5 nm for NETri and 42.9 nm for NEPA. PDI values of NETri hardly changed (Figure 3A), while the PDI of NEPA increased ~1.5-fold even though the formulation maintained its macroscopic characteristics. Although NETri showed the most pronounced increase in Zeta potential, no creaming, phase separation, or coalescence was noticeable. Drug incorporation affected stability as can be observed in Figure 3B. After 90 days, an increase in the zeta potential of PE-NETri (~1.5-fold) was observed. It has been reported that drug encapsulation and release over time might cause changes in the droplet surface structure, resulting in changes in the orientation of the phosphatidylcholine head groups and thus, in the zeta potential [52]. In addition, more pronounced changes in droplet size and PDI of PE-loaded NEPA, but not PE-NETri, was observed, suggesting that PE incorporation affected NEPA stability. Thus, only PE-NETri was further studied.

A preliminary assessment of drug content in NETri was conducted after 30 days of storage at room temperature (maintained by air conditioning set at 25 °C), protected from light (Supplementary Figure S2A). Paclitaxel and elacridar content was higher than 95%, which suggests their stability during the time period investigated. Additionally, paclitaxel and elacridar release from NETri was studied to ensure that, as lipophilic drugs, they would not be retained in the nanoemulsion for long periods of time. Release increased with time (Supplementary Figure S2B), and at 24 h, 78.2 ± 12.4% of paclitaxel and 60.0 ± 11.7% of elacridar were released from NETri. After data fitting, drug release could be better described by the Higuchi’s model (R^2^ > 0.97 and 0.99 for paclitaxel and elacridar, respectively), which is consistent with other studies employing nanoemulsions [53,54,55].

### 2.3. P-Glycoprotein Inhibition Assay

Compounds that perturb membranes, affect ATP binding and/or deplete ATP have been demonstrated to inhibit P-gp-mediated transport [56,57]. Polysorbates and other surfactants can inhibit P-gp because of their effect on membranes, but required concentrations are often high [40,44,58]. As proof of concept that elacridar incorporation improved the ability of NETri to inhibit efflux transports, P-gp ATPase activity was compared after treatment of membranes that express this transporter with NETri and elacridar-loaded-NETri (E-NETri).

**Figure 4:**
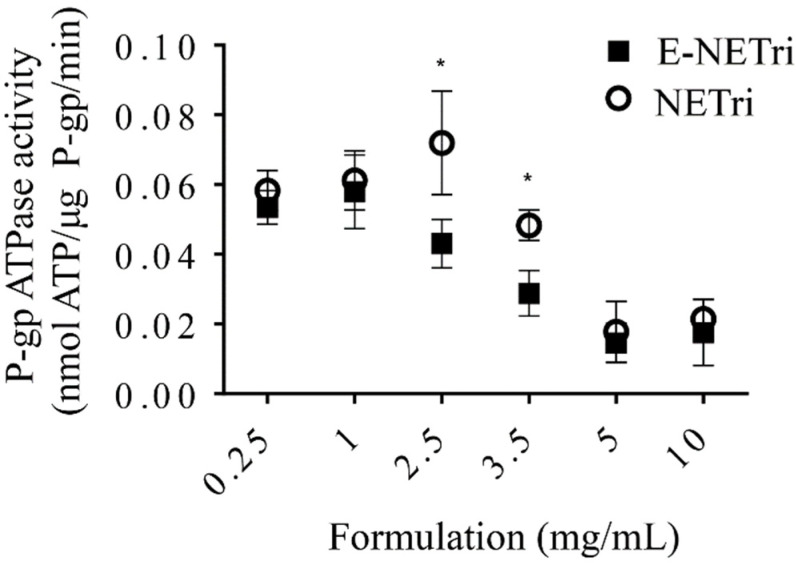
Influence of NE concentration on verapamil-induced P-gp ATPase activity. Sidak’s statistical test showed significant difference between E-NETri compared to NETri (* *p* < 0.05) at 2.5 and 3.5 mg/mL. Data represented as the mean ± SD, of 3–5 samples.

Compared to the unloaded NETri, E-NETri promoted significant reductions on P-gp activity at 2.5 and 3.5 mg/mL (*p* < 0.05, Two-way ANOVA, Sidak’s multiple comparisons post-test) (Figure 4). These results suggest that incorporation of elacridar in NETri potentiated the nanoemulsion ability to inhibit P-gp, resulting in more pronounced transporter inhibitions at lower nanoemulsion concentrations.

### 2.4. Cytotoxicity Evaluation of the Drug-Loaded NETri

Next, the cytotoxic effects of the drug-loaded NETri were investigated on breast cancer cells as monolayers and spheroids. The experiments were conducted to assess whether (i) paclitaxel incorporation in NETri increased its cytotoxic effects compared to the drug solution, since drug incorporation in micro and nanoemulsion affects drug solubility and delivery into cells and tissues [14,59], and (ii) elacridar co-incorporation influenced formulation cytotoxicity.

#### 2.4.1. Cytotoxicity in Cell Monolayers

**Figure 5:**
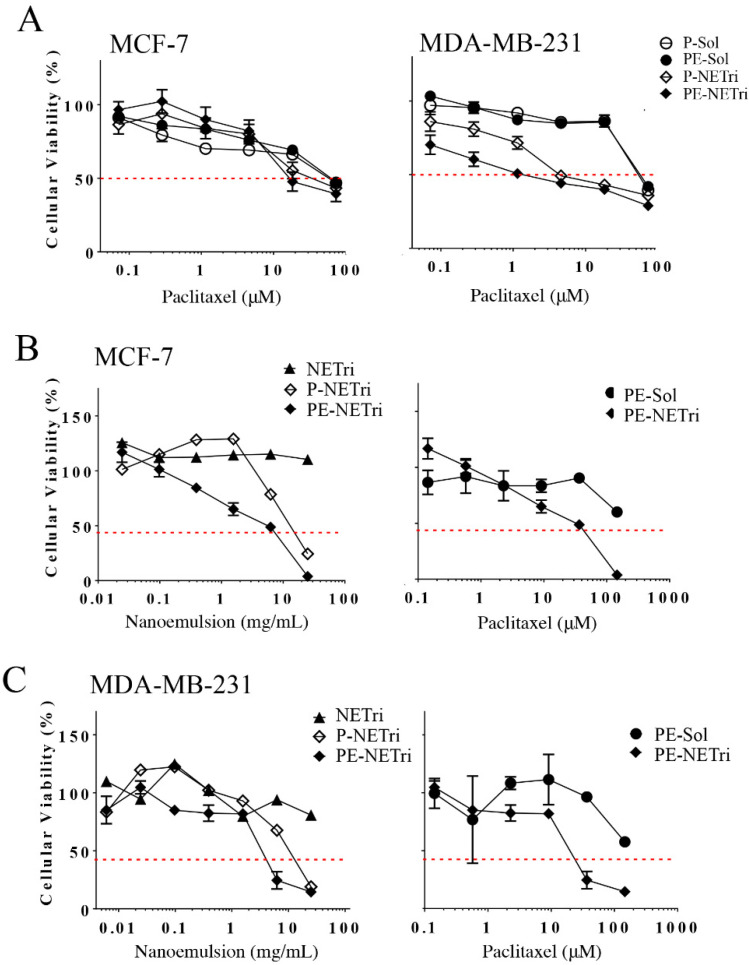
Influence of the concentration of paclitaxel solution (P-Sol), paclitaxel + elacridar solution (PE-Sol), unloaded nanoemulsion (NETri), paclitaxel-loaded (P-NETri), or paclitaxel + elacridar-loaded nanoemulsion (PE-NETri) on the viability of breast cancer cells in 2D and 3D (spheroids) culture. (**A**) Viability of MCF-7 and MDA-MB-231 cells in monolayer (2D culture) after 48 h of treatment. Data are represented as the mean ± SD, *n* = 12–15 in 3–4 separate experiments; (**B**) viability of MCF-7 spheroids after 72 h of treatment, (**C**) viability of MDA-MB-231 spheroids after 72 h of treatment. Data are represented as the mean ± SD, *n* = 3–4. The concentrations of the formulation and paclitaxel are presented in mg/mL and μM, respectively.

Incorporation of paclitaxel in the selected nanoemulsion (P-NETri) increased formulation cytotoxicity (up to 10-fold) compared to the unloaded NETri (Table 2). The cells were not very sensitive to paclitaxel, as demonstrated by IC_50_ values of ~50–60 μM when the drug solution was employed. Similar IC_50_ values of paclitaxel in breast cancer cell lines were described by other groups [60]. Compared to paclitaxel solution (P-Sol), incorporation of paclitaxel in NETri (P-NETri) improved cytotoxicity, although the magnitude of this effect was dependent on cell type (Figure 5). IC_50_ values were reduced ~2.5 to 12.4-fold, with the most pronounced reduction observed in MDA-MB-231 cells. This effect might be related to the higher expression of the CD44 receptors in the triple negative cells compared to MCF-7 cells [33,61], suggesting that the presence of HA in the nanoemulsion might help to improve paclitaxel cytotoxicity in cells that express CD44 receptors.

A comparison of paclitaxel solution with and without elacridar (P-Sol and PE-Sol) indicated that elacridar did not affect cytotoxicity and IC_50_ values pronouncedly in any of the cell lines (Figure 5A and Table 2). On the other hand, co-incorporation of elacridar and paclitaxel in NETri produced 1.6–2.9-fold reductions on the viability of MCF-7 and MDA-MB-231 cells compared to P-NETri, suggesting a possible effect of the NE on elacridar (and not only paclitaxel) delivery. These results indicate that the formulation is a suitable system for co-delivery of paclitaxel and elacridar, increasing paclitaxel cytotoxicity compared to its solution.

#### 2.4.2. Cytotoxicity in Spheroids

Having demonstrated that paclitaxel incorporation in the nanoemulsion improved its cytotoxicity in cell monolayers, the formulation effect on spheroids of MDA-MB-231 and MCF-7 was subsequently assessed. Spheroids have been described to simulate the in vivo cellular microenvironment better than monolayer cultures, justifying their use as 3D models. The unloaded NETri did not promote reductions on the viability of MDA-MB-231 or MCF-7 spheroids to less than 80% (Figure 5B,C and Table 3), not even at the highest concentration tested. This can be justified by the fact that spheroids are more complex structures compared to cell monolayers, with more consistent diffusional barriers. Incorporation of paclitaxel in the nanoemulsion (P-NETri) increased formulation cytotoxicity in both spheroids, and IC_50_ values of 6.6 and 11.1 μM were observed (Table 3). Further increases were observed when elacridar was co-incorporated with paclitaxel, with the most pronounced effect being observed in MDA-MB-231 spheroids (3.2-fold reduction on the formulation IC_50_ compared to P-NETri, Table 3).

**Figure 6:**
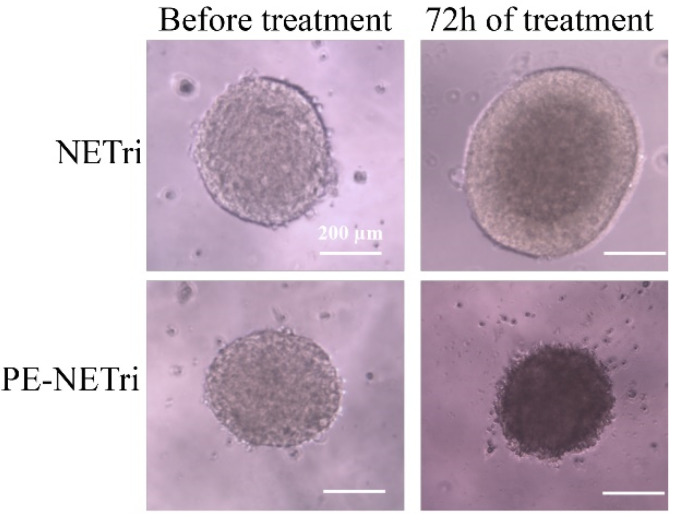
MDA-MB-231 spheroids observed before and after treatment for 72 h with the unloaded NETri or PE-NETri at the IC_50_ value of PE-NETri.

Similar to the unloaded formulation, PE solution did not reduce spheroid viability to 50% or less. A shift of the viability curves to the left, characterizing increased cytotoxicity, was observed upon coincorporation of paclitaxel and elacridar in NETri. Again, the lowest IC_50_ value (19.7 μM) was observed for MDA-MB-231 spheroids (Table 3). As can be observed in Figure 6, the spheroids treated with PE-NETri at its IC50 value became darker and a larger quantity of loose cells and cell debris could be observed in the medium after the 72 h treatment compared to the spheroids before treatment or to those treated with the unloaded NETri (at the same concentration). Similar results were obtained for MCF-7 cells.

### 2.5. In Vivo Mammary Retention of a Fluorescent Marker

To determine the ability of the nanoemulsion to localize compounds in the mammary tissue in vivo after intraductal administration and whether HA presence affected tissue retention, a rhodamine-loaded NE was employed.

**Figure 7:**
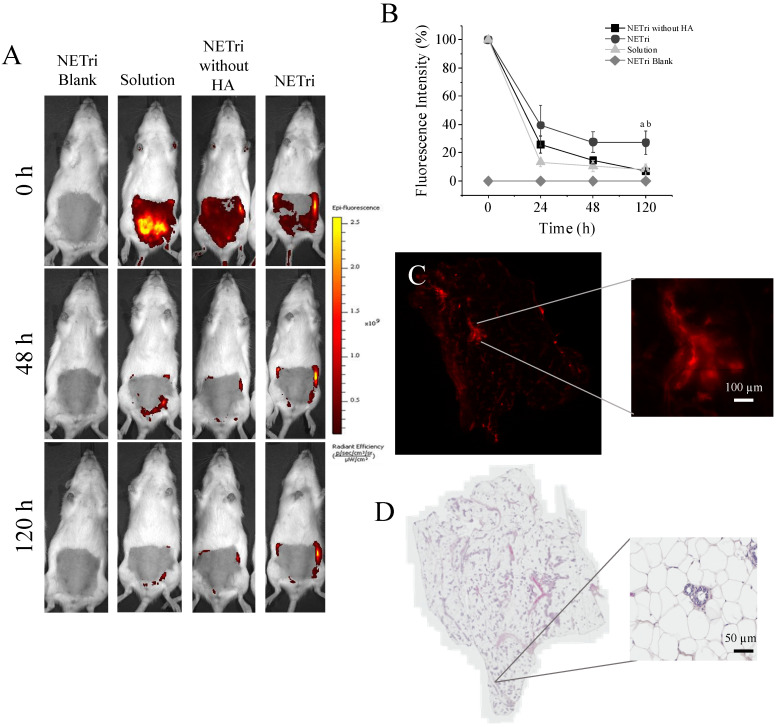
In vivo mammary retention of the fluorescent marker rhodamine over 120 h after intraductal (ID) administration and histological changes. (**A**) Representative images of the fluorescence signal in female rats treated with 0.5% rhodamine solution, NETri without HA and NETri loaded with 0.5% rhodamine; unloaded NETri was employed for autofluorescence assessment. (**B**) Decay of fluorescence intensity along 120 h after ID administration. Data are represented as the mean ± SD. Tukey’s statistical test (*n* = 3–4) showed significant difference between NETri compared to the solution (a = *p* < 0.01) and NETri compared to the NETri without HA (b = *p* < 0.01). (**C**) Fluorescent signal remaining in the breast tissue after 120 h of ID administration of NETri containing 0.5% rhodamine. The zoom shows a ductal tree with its alveoli, suggesting that the fluorescent signal came from inside the ducts. Bar = 100 µm. (**D**) Representative image of the mammary tissue after ID administration of the NETri obtained by optical microscopy. Bar = 100 µm.

The retention of rhodamine in the mammary tissue was monitored for 120 h. The unloaded (rhodamine-free) NETri did not result in any fluorescent staining, demonstrating that signals arise from the presence of rhodamine in the tissue (Figure 7). Intraductal administration of rhodamine solution, rhodamine-loaded NETri (that contains HA), or rhodamine-loaded nanoemulsion without HA led to fluorescent staining of the mammary tissue at the day of injection. Independent of the type of treatment, staining was reduced during 5 days, but reduction was more pronounced using the solution than NETri (Figure 7A). More specifically, staining was reduced to 35 and 10% of the initial intensity using rhodamine-loaded NETri and rhodamine solution, respectively, resulting in over three-fold higher staining signal using NETri (*p* < 0.05) at 120 h (Figure 7B). These results suggest the benefit of associating intraductal delivery and bioadhesive nanoemulsions to prolong mammary retention. Interestingly, staining resulting from administration of NETri without HA was similar to the solution of the fluorochrome (*p* > 0.05, Two-way ANOVA and post hoc Tuckey) and inferior to NETri (that contains HA at 0.25%), reinforcing the role of HA to improve tissue retention and its importance in the formulation.

Even though the intensity of rhodamine signal decayed after 5 days, when we removed the mammary tissue and analyzed tissue staining under a fluorescence microscope, the fluorochrome was still present in the tissue, mainly in the ducts and adjacent tissues. Signal of rhodamine was still intense in the NE-HA group (Figure 7C).

None of the formulations caused inflammatory signs in the mammary tissue, preserving the characteristics of the ducts and tissue organization, including the epithelium, adipose tissue with unilocular adipocytes and connective tissue (stroma) that surrounds the ducts, ductules (smaller ducts), and alveoli [62]. The adipose tissue presented lymph nodes, blood vessels, nerves and smooth muscle fibers [62], and no abnormal presence of lymphocytes or other signs of infiltration of inflammatory cells. The lumen of the ducts and alveoli were also clean of secretion or cells, indicating no abnormality or lesion (Figure 7D). The absence of changes in weight, locomotion, and leukocyte count in the animals (Supplementary Table S1) support the potential safety of the formulation when administered through the intraductal route, although further evaluation of histological changes in the mammary tissue and other biochemical parameters (such as liver function) after longer treatment periods would be necessary to ensure safety.

## 3. Discussion

The chemotherapeutic agent paclitaxel has been widely used for treatment of solid tumors, but its ability to induce a variety of severe adverse effects remains a limitation that motivates the search for new delivery systems and routes of administration to maintain efficacy and limit systemic toxicity [63,64]. In this study, we aimed at developing a nanoemulsion for co-incorporation and intraductal delivery of paclitaxel and elacridar. The composition was carefully selected and optimized in this study to enable the obtainment of multiple properties that are relevant for intraductal administration and breast cancer treatment.

Cell culture studies helped us to understand the impact of formulation composition on its cytotoxicity. We demonstrated that tributyrin and PA at 0.5–1% increased formulation cytotoxicity, but this effect was observed only in MCF-7 cells. Cytotoxicity enhancement mediated by tributyrin was also observed by Migotto et al. in this cell line, which caused a reduction in the IC_50_ of nanoemulsions of more than 80% [8]. The present study confirms the cytotoxicity potential of the triglyceride, but also provides evidence that this effect is observed when it is incorporated in the NE at eight-fold lower concentrations. As to PA, Yeruva and collaborators demonstrated that PA treatment reduced the viability and increased apoptosis in 33.7% and 12.6% in MDA-MB-231 and MCF-7, respectively, possibly by the induction in G0/G1 arrest, sensitizing cells and reducing IC_50_ values of cisplatin [65]. Increases of PA content in the NE might be relevant to further improve cytotoxicity in MDA-MB-213 cells, since the reported IC_50_ of PA to inhibit the proliferation of these cells was 1.5 mM. Only at the highest NE concentration (50 mg/mL), PA content in the cell culture medium would be closer to this value.

Despite the similar cytotoxicity of tributyrin and PA-containing nanoemulsions, NETri was more stable upon drug loading. To the best of our knowledge, the relationship between presence of PA and reduction of NE stability has not been previously reported. Feng et al. observed that some D-limonene-loaded nanoemulsions demonstrated instability (creaming, larger droplets and high Turbiscan Stability Index) depending on the type of surfactant [66]. As a limonene constituent, perillyl alcohol might require other types of surfactant systems to form stable nanoemulsions. It is also worth considering the role of drug localization in the nanoemulsion, since it has been demonstrated that drug loading into phospholipid-stabilized emulsified systems and the compartment where they localize can influence stability [50]. As previously mentioned, lipophilic drugs (like paclitaxel and elacridar) can be localized in various compartments of the nanoemulsion: dissolved/entrapped into the oil droplets, in the surfactant interface, and dispersed/dissolved in the aqueous phase [49,50,67]. Due to the small droplet size and high interface-to-core ratio, drug localization at interfaces might influence the surfactant function, and thus, stability of the droplets [50]. Since PA is less lipophilic than tributyrin (logP of 1.9 compared to a predicted logP of 2.9 for tributyrin) [68], it is reasonable to suggest a difference in their ability to dissolve paclitaxel and elacridar, which might favor drug incorporation at the surfactant interface in the presence of PA, affecting surfactant function and nanoemulsion stability. In addition, since nanoemulsions are prone to Ostwald ripening and reducing aqueous solubility of the oil phase helps to prevent this process [69], the higher lipophilicity of tributyrin might have rendered NETri more stable.

Paclitaxel incorporation in NETri improved its cytotoxicity. Paclitaxel is considered an effective chemotherapeutic drug, employed as cornerstone in early or advanced breast cancer treatment despite its many toxic effects, and thus, nanocarriers have been frequently studied to improve cytotoxicity and cancer cell targeting. For example, Bernabeu and collaborators reported that paclitaxel incorporation in nanoparticles functionalized with TPGS-b-PCL reduced its IC_50_ more than 60% [60]. Pepe et al. reported a 2-fold reduction on the IC_50_ value in basal cell carcinoma cells when the drug was incorporated in a microemulsion [59]. Carvalho et al. demonstrated that paclitaxel incorporation in a nanoemulsion reduced its IC_50_ by 4-fold in SK-MEL-19 cell [16]. A study by Bu and collaborators also showed that a nanoemulsion based on TPGS, Tween 80, and medium chain triglyceride reduced the IC_50_ in MCF-7/ADR cells by 18.9-fold compared to the drug solution [70]. The cytotoxicity enhancement mediated by nanoemulsified carriers has been associated with an improved solubilization of lipophilic drugs in the culture medium and delivery to cells and tissues [16,71]. The improved delivery might be attributed to more efficient passive diffusion—due to the presence of surfactants and other penetration enhancers as nanoemulsion components—and/or specialized mechanisms [72,73]. Previous studies suggest that phosphatidylcholine and surfactants (such as polysorbates) might act as absorption enhancers, altering the organization and permeability of biological barriers and improving drug diffusion across them [72,74,75,76]. Presence of polysorbates and poloxamers in nanocarriers has been suggested to induce endocytosis across the blood–brain barrier due to association with lipoproteins and other biological molecules [77].

Additionally, hyaluronic acid has been described as a ligand of CD44, being able to induce cell internalization by CD44-mediated endocytosis [78]. Using differential scanning calorimetry, we have previously demonstrated that HA incorporation increased the glass transition temperature (T_g_’) in nanoemulsions from −45 to −36 °C, the melting peak temperature (T_peak_) from −0.8 to +1.4 °C, and enthalpy of fusion (∆H_fus_) from 214.7 to 243.0 J/g, which suggest its ability to affect the mobility of water at the vicinity of interfaces [12]. These results indicate that at least part of the polysaccharide content can be located at or in close proximity to the interface. Although the types of interactions between HA and surfactants forming these nanoemulsions were not further investigated, previous studies employing cationic micelles reported that hyaluronan chains do not penetrate into the interior of micelles formed with decyl- and dodecyltrimethyl-ammonium bromides, and charged groups of hyaluronic acid might act as counterions [79,80]. In addition, hydrophilic headgroups of non-ionic and anionic surfactants (such as sodium dodecyl sulfonate) were attracted by the domains formed by the hydroxyl groups of hyaluronate [80]. It was also suggested that the hydrophobic surface of phospholipid-based aggregates can potentially interact with HA by binding the hydrophobic regions along the HA chain [81,82].

We also demonstrated that paclitaxel combination with elacridar in the nanoemulsion, but not as a solution, increased its cytotoxicity, which further corroborates the benefit of nanoemulsified systems for solubilization and delivery of lipophilic compounds such as paclitaxel and elacridar. Considering that elacridar inclusion in the nanoemulsion did not preclude its ability to inhibit P-gp, the increment in paclitaxel cytotoxicity might be related to an improved cell uptake of elacridar and inhibition of efflux transporters. MCF-7 and MDA-MB-231 cells have been demonstrated to express P-gp, although at much lower levels than resistant cells, suggesting a possible role of elacridar in preventing efflux [83,84]. The benefits of nanocarriers co-loaded with paclitaxel and elacridar to increase cytotoxicity, inhibit efflux, and reverse multidrug resistance have been demonstrated in other studies. For example, Tonbul et al. reported that while paclitaxel-loaded nanoparticles alone at 50 µM displayed almost no cytotoxic effect in EMT6/AR1.0 mouse mammary tumor cell line, the cellular viability decreased in approximately 77% when elacridar (100 nM) was co-encapsulated [85]. Future studies will assess formulation cytotoxicity in paclitaxel-resistant breast cancer cells (our group has been working on the development of these cells). Clinically, the potential in augmenting the effectiveness of chemotherapeutic drugs by their association with inhibitors of efflux transporters is highly desirable, but since transporters are not exclusively expressed in tumors, enhancing paclitaxel’s cytotoxic effect could culminate in a proportional enhancement of systemic adverse effects and toxicity [86]. Local administration into the mammary ducts can overcome this problem.

Hyaluronic acid (HA) was included in the nanoemulsion primarily because of its bioadhesive properties [12,87,88], but this natural anionic polysaccharide also plays other roles. As already mentioned, it is a ligand of CD44; because it is overexpressed in several types of cancer, it is a potential target to increase the specificity of treatments toward tumor cells [12,89]. We demonstrated in vivo that HA presence in the nanoemulsion was essential to prolong the local retention of rhodamine, since the NE without HA promoted similar retention as the solution. Our results are in consonance with Barbault-Foucher et al., who demonstrated the mucoadhesive potential of HA in an ophthalmic drug delivery system based on poly-ε-caprolactone nanospheres possibly due to its non-covalent attachment to the precorneal mucin layer [87]. Mucins are important for protection and lubrication of epithelium-lined ducts [90], and the fact they are present in the mammary ducts represents an important feature of the route that justifies the design of bioadhesive nanocarriers. We have previously demonstrated that HA-modified nanoemulsions were able to prolong mammary tissue retention of a hydrophilic probe [12], but to our knowledge, this is the first evidence that HA presence in the nanoemulsion was indeed necessary for this effect and that a four-fold lower HA concentration was sufficient. The formulation did not change the histological characteristics of the mammary tissue, demonstrating that it does not promote local adverse reactions during the time period investigated.

## 4. Materials and Methods

### 4.1. Materials

Tricaprylin was kindly supplied by Croda Health Care (Edison, NJ, USA). Soy phosphatidylcholine (PC) and 2-dipalmitoyl-sn-glycero-3-phosphoethanolamine (DPPE) were obtained from Avanti Polar Lipids (Alabaster, AL, USA), and propylene glycol and glycerol were purchased from Synth (São Paulo, SP, Brazil). Paclitaxel and elacridar were obtained from Cayman Chemical Company (Ann Arbor, MI, USA). Tributyrin (Tri), perillyl alcohol (PA), tetrazolium dye 3-(4,5-dimethylthiazol-2-yl)-2,5-diphenyltetrazolium bromide (MTT), and polysorbate 80 were purchased from Sigma (St Louis, MO, USA). Other specific reagents are described along with the respective methodology. Ultrapure water was employed unless stated.

### 4.2. Nanoemulsion Development

Nanoemulsions were produced by combining the oil phase and surfactant mixture (PC:DPPE:polysorbate 80:propylene glycol:glycerol 3:0.2:1:0.5:0.45, *w/w/w/w/w*) at 1:1 (*w/w*, composing 20% of the formulation). We employed phospholipids in the surfactant blend to reduce surfactant-based irritation and because DPPE has been demonstrated to increase nanoparticle uptake by cancer cells [91,92]. Subsequently, the aqueous phase (80% of the formulation) was heated to 40 °C and added to the mixture under vortex mixing for 30 s. Subsequently, the formulations were sonicated in an ice bath, with a regimen of 58 s on and 30 s off for 10 min (VCX-500, Sonics, Newtown, CT, USA).

Particle diameter, polydispersity index (PDI), and zeta potential were determined in NanoZS90 (Zetasizer, Malvern, UK) equipment after nanoemulsion dilution with water at 1:100 (*w/w*). The rheological behavior of the formulations was investigated using an R/S Plus controlled stress rheometer with the RC75-1 geometry (Brookfield Engineering Laboratories, Middleboro, MA, USA) and controlled temperature set at 25 °C. The experiments were performed with shear rates varying up to 2000 s^−1^ [48].

This nanoemulsion preparation protocol underwent two variations for assessment of the influence of aqueous and oil phase composition on the nanoemulsion characteristics, as described in subsequent subsections.

#### 4.2.1. Influence of Oil Phase on NE Formation

To assess the influence of tributyrin (Tri) and perillyl alcohol (PA) on nanoemulsion characteristics, three types of oil phase were tested: tricaprylin and tricaprylin containing either perillyl alcohol or tributyrin (ranging from 0.5 to 5% as final concentration in the nanoemulsion). The oil phase was combined with the surfactant mixture before aqueous phase addition and sonication. Homogeneous formulations were kept at room temperature, protected from light for up to 7 days to assess whether they remained preliminarily stable (no signs of creaming, phase separation or aggregation) before further testing.

#### 4.2.2. Influence of the Aqueous Phase on NE Formation

The effect of adding hyaluronic acid (HA, low molecular weight, 10 KDa, Lifecore Biomedical, Chaska, MA, USA) on the NE characteristics was assed next. It was dissolved in PBS prior to aqueous phase incorporation to obtain final concentrations of 0.125, 0.25, and 0.5%. The formulations were sonicated as described. Homogeneous formulations were kept at room temperature, protected from light for 7 days to assess whether they remained stable before further testing.

### 4.3. Drug Incorporation, Short-Term Stability and Release

Paclitaxel and elacridar were dissolved in selected oil phase:surfactant mixtures prior to aqueous phase addition. Two concentrations of elacridar (0.1% and 0.07%) and paclitaxel (0.5% and 1%) were tested based on previous studies of drug solubility in micro and nanoemulsions [16,27,59] and their influence on the physicochemical characteristics was assessed. Droplet diameter, polydispersity index (PDI), and zeta potential, were analyzed using NanoZS90 (Zetasizer, Malvern, UK) equipment after nanoemulsions dilution with water at 1:100 (*w/w*).

Based on their characteristics, selected unloaded NEs and those containing elacridar (0.07% *w/w*, 1.2 mM) and paclitaxel (0.5% *w/w*, 5.9 mM) were subjected to a short-term stability test. Formulations (3 batches) were kept at room temperature (maintained by air conditioning set at 25 °C) protected from light for 90 days and subjected to visual and microscopic inspection (Leica, Wetzlar, Germany) for signs of creaming, phase separation or aggregation. Furthermore, droplet size, PDI and zeta potential were assessed as previously described.

To evaluate whether paclitaxel and elacridar were released from the selected NE, the formulation was placed in the receiver compartment of Franz diffusion cells in small dialysis bags (14,000 Da cutoff, Sigma-Aldrich, St. Louis, MO, USA) in phosphate buffered saline (PBS) + 1% polysorbate 80, pH 7.4, at 37 °C under stirring (150 rpm) as previously described [93]. Aliquots of the receptor phase were then withdrawn (0.25 mL) at predetermined time periods (3–24 h) and the samples were analyzed by HPLC using a Shimadzu HPLC system equipped with a Phenomenex C18 column [16,59]. Paclitaxel was assayed at 228 nm using mobile phase composed of 55:45 (*v/v*) acetonitrile:water at a flow rate of 1.0 mL/min, with injection of 20 µL at room temperature (25 °C). Elacridar was assayed using mobile phase composed of acetonitrile:water (60:40, *v/v*) at 1 mL/min and UV detection at 249 nm, with injection of 20 µL at room temperature (25 °C). Calibration curves of paclitaxel (0.2–100 µg/mL, R^2^ > 0.995) or elacridar (2–20 µg/mL, R^2^ > 0.993) prepared in methanol were used for drug quantification. Data were fitted to the zero-order kinetics (Q_t_ = Q_0_ + K_0_ t); Higuchi kinetics (Q = K_h_t^1/2^); and first-order kinetics (log Q_t_ = −K_t_/2303 + log Q_0_), in which Q_t_ represents the absolute amount of drug released in t (time in hours), Q_0_ is the initial amount of drug in the solution, K_0_ is a zero-order kinetic constant, and K_h_ is the Higuchi dissolution constant [93].

We also assessed drug content after 30 days of storage of the selected nanoemulsion (NETri) to estimate whether the drugs would be degraded after short-term storage (30 days) at room temperature (maintained by air conditioning set at 25 °C) protected from light. The drugs were quantified by HPLC after NE dilutions (triplicates, different dilutions for paclitaxel and elacridar since the former was incorporated at a higher amount) with methanol to yield theoretical drug concentrations of 7 (elacridar) or 10 (paclitaxel) μg/mL.

### 4.4. Cytotoxicity Assays: 2D and 3D Models

#### 4.4.1. Cytotoxicity Evaluation in Cell Monolayers (2D Model)

Because the choice of components influences drug delivery and cytotoxicity [44,94], the effect of nanoemulsion composition and drug incorporation on the viability of two breast cancer cell lines (MCF-7 and MDA-MB-231, ATCC, Manassas, VA, USA) was studied.

The cells were maintained with DMEM/F12 culture medium supplemented with 10% fetal bovine serum and antibiotics at 37 °C in a 5% CO_2_ atmosphere. When they reached approximately 80% of confluence, cells were subjected to trypsinization and transferred to 96-well culture plates at a concentration of 10,000 cells/well. Cells were treated with the selected unloaded NEs, drug-loaded formulations or paclitaxel solution for 48 h [95,96] at concentrations ranging from 0.006 to 25 mg/mL of formulations and 0.07 to 73.2 µM of paclitaxel. Cell viability was evaluated by MTT as proposed by Mosmann [97]. Untreated cells and those treated with PBS (vehicle for the NE), doxorubicin, and DMSO (solvent for doxorubicin) were employed as controls. The concentration necessary to reduce cell viability to 50% (IC_50_) was confirmed using trypan blue. Briefly, cells were treated with the nanoemulsions at the IC_50_ determined by MTT; following treatment, they were stained with 0.4% trypan blue (1:1 *v/v*) and counted in Neubauer’s chamber. Cells stained by trypan blue were considered nonviable.

#### 4.4.2. Cytotoxicity Evaluation in Spheroids (3D Model)

Spheroids of MCF-7 and MDA-MB-231 were obtained using the liquid overlay technique, which does not allow adhesion to the plate surface [10,93]. Accordingly, 96-well microplates were prepared with 50 μL of 1% agarose solution in each well [93], 5 × 10^3^ cells/well were seeded, the plate was centrifuged at 1000 RPM for 7 min, and incubated at 37 °C in a 5% CO_2_ incubator.

The spheroids were treated with serial dilutions (starting at 73 µM) of paclitaxel solution, unloaded NE (NE without paclitaxel or elacridar), NE with paclitaxel, or NE with paclitaxel and elacridar for 72 h. Spheroids were subsequently submitted to viability assessment (CellTiterGlo^®^ 3D, Promega, Madison, WI, USA) employing luminescent measurement of ATP levels, followed by determination of absorbance in a luminescence reader (560 nm) as per manufacturer’s instructions.

### 4.5. Glycoprotein-P Inhibition Assay

To assess whether elacridar incorporation affected the NE ability to inhibit ATP hydrolysis and P-gp-mediated transport, the transporter activity was assessed using the Pro-Glo™ Assay (Promega, Madison, WI, USA) and standardized recombinant P-gp membranes as previously described [27,44]. The nanoemulsions with and without elacridar were tested at final concentrations ranging from 0.25 to 10 mg/mL. P-gp-expressing membranes were stimulated by the L-type calcium channel blocker verapamil, while sodium orthovanadate (Na_3_VO_4_) was used as control (background control and a measure of P-gp-independent ATPase activity) for P-gp ATPase inhibition. The P-gp inhibitory effects of the NEs were determined using a luminometer and the data were converted to P-gp ATPase activity based on a calibration curve obtained using ATP standards at 0.375–3 mM. For reference, the basal activity, calculated based on ATP levels in membranes treated with Na_3_VO_4_and untreated membranes, was 0.07 nmol consumed ATP/µg P-gp/minute.

### 4.6. In Vivo Mammary Retention of a Fluorescent Marker Mediated by the Selected Nanoemulsion

Female Wistar rats (7–8 weeks) were previously housed with free access to food and water. The animal room was maintained under the light-dark cycle (12:12 h) with temperature at 22–23 °C. All experiments were conducted according to the guidelines of the National Council for Animal Experimentation (CONCEA) and approved by the Animal Care and Use Committee of the Institute of Biomedical Sciences of the University of São Paulo (protocol number #69/2016, São Paulo, Brazil). Before treatment, rats were anesthetized by inhalation of isoflurane (2–2.5%) to remove abdomen hair with the aid of VEET^®^ cream, the area was gently rubbed with alcohol-soaked cotton to reveal the duct orifice [8,12]. Three pairs of nipples were selected according to their ease of access, and the nanoemulsions containing rhodamine (0.5%), obtained by dissolving the dye (0.5%, *w/w*) in the surfactant:oil phase mixture, were intraductally administered (20 µL) with a 33G needle (Hamilton, Bonaduz, Switzerland) coupled to a 0.1 mL syringe. The in vivo fluorescence intensity was evaluated using the IVIS Spectrum system (Perkin-Elmer Life Sciences, Waltham, MA, USA located at CEFAP-ICB-USP) for 120 h.

Fluorescence intensity at the mammary tissue was monitored using an absorption filter at 465–540 nm, with an exposure time of 0.5 s and a binning value of 8–2. After the experiment, the mammary tissue was removed and either (i) fixed for histology to assess NE-mediated changes on the tissue organization or (ii) embedded in optimum cutting temperature compound to obtain 9 μm section using cryostat (Leica CM 1850 UV) to evaluate the permanence of rhodamine-loaded NE fluorescent signal in the mammary ducts. Tissues were then observed on Axioscan 7 (Zeiss, Germany).

### 4.7. Statistical Analyses

The results were statistically analyzed using ANOVA test followed by Tukey or Sidak’s post-hoc test (GraphPad Prism software, San Diego, CA, USA). Values were considered significantly different when *p* < 0.05.

## 5. Conclusions

In this study, we optimized nanoemulsion composition for co-incorporation of paclitaxel and elacridar and provided evidence that the selected nanocarrier (i) presented suitable characteristics and short-term stability, and (ii) increased the cytotoxicity of paclitaxel, which was further improved by the presence of elacridar in 2D and 3D models. Furthermore, elacridar reduced the concentration of nanoemulsion necessary to inhibit P-gp ATPase activity. Nanoemulsion modification with HA was essential to prolong the in vivo retention of rhodamine (incorporated in NETri). These results demonstrate that the nanoemulsion developed here can deliver elacridar and paclitaxel to cancer cells and enhance the cytotoxic effect of paclitaxel even in triple negative breast cancer cell lines. The main relevance of this finding is that treatment options for triple negative breast cancer are scarce, opening other venues for investigation of new therapeutic strategies for the disease.

## 6. Patents

The authors filed a patent application related to this study in Brazil (BR 10 2017 015966 3).

## Figures and Tables

**Table 1:** Influence of the type of oil phase on the droplet size, PDI and zeta potential of nanoemulsions without HA, and influence of the concentration of HA, paclitaxel (P) and elacridar (E) on the physicochemical characteristics of nanoemulsions containing perillyl alcohol (PA) or tributyrin (Tri).

Nanoemulsion	Oil Phase/Drug Concentration	Size (d.nm)	PDI	Zeta Potential (mV)
NE (no HA)	Tricaprylin	164.3 ± 7.8	0.178 ± 0.02	−23.8 ± 2.8
NETri (no HA)	Tricaprylin + tributyrin (1%)	100.89 ± 1.1	0.114 ± 0.04	−41.7 ± 1.8
	Tricaprylin + tributyrin (2.5%)	-	-	-
NEPA (no HA)	Tricaprylin + PA (0.5%)	175.1 ± 1.8	0.204 ± 0.11	−27.2 ± 1.2
	Tricaprylin + PA (1%)	139.4 ± 0.9	0.192 ± 0.01	−12.1 ± 0.7
	Tricaprylin + PA (2.5%)	155.2 ± 1.7	0.170 ± 0.04	−17.7 ± 2.2
	Tricaprylin + PA (5%)	-	-	-
E-NETri (with 0.25% HA)	Tricaprylin + tributyrin (1%) 0.1% E	608.1 ± 0.4	0.327 ± 0.01	−19.9 ± 3.1
	Tricaprylin + tributyrin (1%) (0.07% E)	202.9 ± 0.7	0.197 ± 0.03	−17.5 ± 1.8
PE-NETri (with 0.25% HA)	Tricaprylin + tributyrin (1%) 0.07% E + 1% P	228.9 ± 1.0	0.459 ± 0.01	−19.0 ± 5.9
	Tricaprylin + tributyrin (1%) 0.07% E + 0.5% P	114.6 ± 1.2	0.292 ± 0.01	−32.4 ± 0.28
E-NEPA (with 0.25% HA)	Tricaprylin + PA (0.5%) 0.1% E	329.2 ± 8.7	0.216 ± 0.02	−11.4 ± 0.2
	Tricaprylin + PA (0.5%) 0.07% E	138.7 ± 1.9	0.183 ± 0.11	−15.1 ± 1.5
PE-NEPA (with 0.25% HA)	Tricaprylin + PA (0.5%) (0.07% E + 1% P)	216.0 ± 14.8	0.413 ± 0.06	−34.2 ± 6.7
	Tricaprylin + PA (0.5%) (0.07% E + 0.5% P)	134.2 ± 3.8	0.293 ± 0.01	−26.0 ± 1.4

**Table 2:** Values of IC_50_ related to MCF-7 and MDA-MB-231 cell lines.

	MCF-7		MDA-MB-231	
IC_50_	mg/mL of NE	µM of P	mg/mL of NE	µM of P
U-NE without HA	-	-	15.5	-
U-NE	-	-	6.6	-
NETri	9.7	-	8.2	-
NEPA	12.6	-	9.7	-
P-Sol	-	78.8	-	61.8
PE-Sol	-	66.0	-	62.4
P-NETri	4.7	27.4	0.9	5.0
PE-NETri	2.9	17.1	0.3	1.7

**Table 3:** Values of IC_50_ related to 3D cell culture of MCF-7 and MDA-MB-231.

	MCF-7		MDA-MB-231	
IC_50_	mg/mL of NE	µM of P	mg/mL of NE	µM of P
NETri	-	-	-	-
PE-Sol	-	-	-	-
P-NETri	6.6	38.5	11.1	64.8
PE-NETri	3.9	23.1	3.4	19.7

## Data Availability

Data is contained within the article.

## Supplementary Materials

The following supporting information can be downloaded at: https://www.mdpi.com/article/10.3390/ph15091110/s1. Figure S1: Size distribution obtained by dynamic light scattering corresponding to NETri containing elacridar (E) at 0.07% or 0.1% (upper panel) and NETri containing elacridar (0.07%) + paclitaxel (P) at 0.5% or 1% (lower panel). The size of E-NETri containing elacridar at 0.1% was higher than expected (608.1 nm), and the 0.07% concentration was selected. The NE containing paclitaxel at 1% displayed two peaks and PDI value above 0.4, indicating polydisperse samples. PE-NETri with 0.5% paclitaxel was selected to further studies. Figure S2. Paclitaxel release from the P-NETri. Cumulative release of paclitaxel at 0.5% in the formulation as a function of time. At 3 h, more than 36% of the drug in the NE diffused across the membrane, while release in 72 h reached 78.2%. Data represented as mean ± standard deviation, *n* = 4. Table S1: White Blood Cell (WBC) count in female rats 5 days after intraductal administration.
