# Supplementary material for: Phosphatidylcholine-Based Nanoemulsions for Paclitaxel and a P-Glycoprotein Inhibitor Delivery and Breast Cancer Intraductal Treatment
Source: Pharmaceuticals (Basel). 2022 Sep 6;15(9):1110. doi: 10.3390/ph15091110 (PMC9503599; doi:10.3390/ph15091110)
Supplement: Supplementary file 1 [file pharmaceuticals-15-01110-s001.zip › pharmaceuticals-1854656-supplementary.pdf]

# Supplementary materials

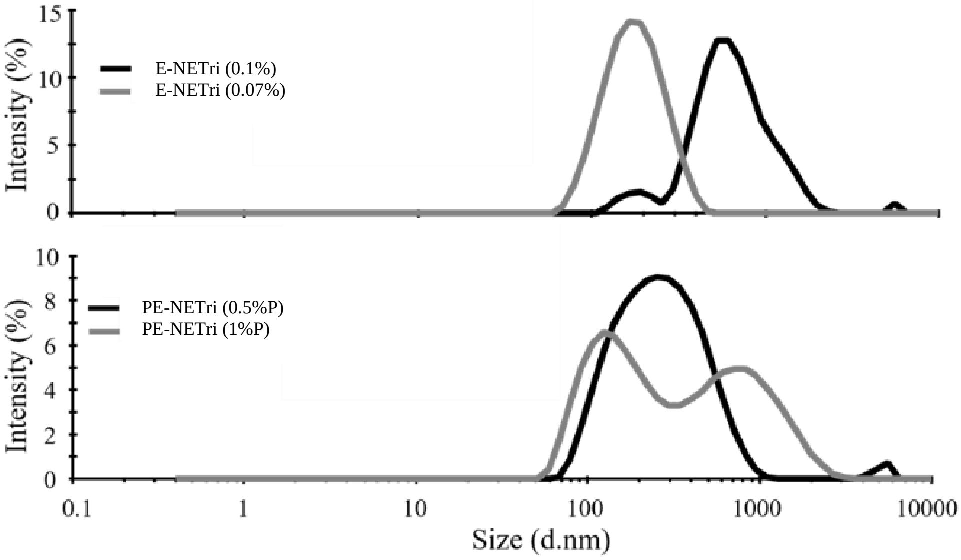


**Figure S1.** Size distribution obtained by dynamic light scattering corresponding to NETri containing elacridar (E) at 0.07% or 0.1% (upper panel) and NETri containing elacridar (0.07%) + paclitaxel (P) at 0.5% or 1% (lower panel). The size of E-NETri containing elacridar at 0.1% was higher than expected (608.1 nm), and the 0.07% concentration was selected. The NE containing paclitaxel at 1% displayed two peaks and PDI value above 0.4, indicating polydisperse samples. PE-NETri with 0.5% paclitaxel was selected to further studies.

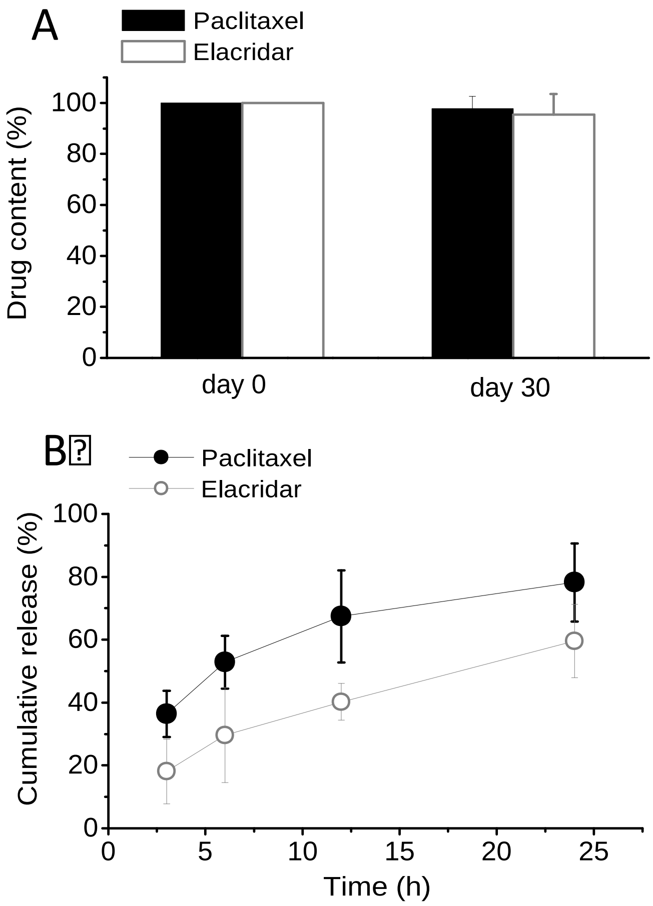


**Figure S2.** Paclitaxel and elacridar content and release over time. A: Content of paclitaxel and elacridar in NETri after 30 days of storage at room temperature protected form light. B: Cumulative release of paclitaxel and elacridar as a function of time. Data represented as mean ± standard deviation, n = 3-4.

**Table S1.** White Blood Cell (WBC) count in female rats 5 days after intraductal administration.

| | **Neutrophil (%)** | | **Lymphocyte (%)** | | **Monocyte (%)** | | **Eosinophil (%)** | | **Basophil (%)** | |
|---|---|---|---|---|---|---|---|---|---|---|
| | Average | SD | Average | SD | Average | SD | Average | SD | Average | SD |
| **ID NETri without HA** | 22 | 2.91548 | 73.2 | 3.563706 | 4.2 | 0.83666 | 0.4 | 0.54772 | 0.2 | 0.44721 |
| **ID NETri** | 21.00 | 1.73205 | 72.33 | 2.081666 | 5.67 | 1.527525 | 0.33 | 0.57735 | - | - |
| **ID Solution** | 21.0 | - | 75.0 | - | 2.0 | - | 2.0 | - | - | - |
| **Reference** | 5.0 - 27.1 | - | 66.8 - 91.3 | - | 0.7 - 4.4 | - | 0.5 - 4.6 | - | 0.0 - 0.5 | - |
